# High rates of midazolam injection among drug users in Bangkok, Thailand

**DOI:** 10.1186/1477-7517-7-7

**Published:** 2010-03-26

**Authors:** Thomas Kerr, Niyada Kiatying-Angsulee, Nadia Fairbairn, Kanna Hayashi, Paisan Suwannawong, Karyn Kaplan, Calvin Lai, Evan Wood

**Affiliations:** 1 British Columbia Centre for Excellence in HIV/AIDS, St Paul's Hospital, Vancouver, Canada; 2 Department of Medicine, University of British Columbia, Vancouver, Canada; 3 Social Pharmacy Research Unit, Chulalongkorn University, Bangkok, Thailand; 4 Thai AIDS Treatment Action Group, Bangkok, Thailand

## Abstract

**Background:**

Reports from Thailand suggest that a growing number of people who inject drugs (IDU) are now injecting midazolam, a legal benzodiazepine with potent amnestic and ventilatory depressant effects. We therefore sought to examine midazolam injection among a community-recruited sample of Thai IDU.

**Methods:**

We examined the prevalence and correlates of midazolam injection among 252 IDU participating in the Mitsampan Community Research Project, Bangkok, using multivariate logistic regression. We also examined the use of midazolam in combination with other drugs.

**Results:**

252 IDU participated in this study, including 66 (26.2%) women. In total, 170 (67.5%) participants reported ever having injected midazolam, and 144 (57.1%) reported daily midazolam injection in the past six months. In multivariate analyses, a history of midazolam injection was independently associated with using drugs in combination (adjusted odds ratio [AOR] = 5.86; 95% confidence interval [CI]: 2.96-11.60), younger age (AOR = 0.43; 95%CI: 0.22-0.83), having a history of methadone treatment (AOR = 3.12, 95%CI: 1.55-6.90), and binge drug use (AOR = 2.25, 95%CI: 1.09-4.63). The drugs most commonly used in combination with midazolam were heroin (72.3%) and yaba (methamphetamine) (30.5%).

**Conclusion:**

We observed a high rate of midazolam injection among Thai IDU. Midazolam injection was strongly associated with polysubstance use and binge drug use, and was most commonly used in combination with both opiates and methamphetamines. Our findings suggest that midazolam injection has become increasingly common within Thailand. Evidence-based approaches for reducing harms associated with midazolam injection are needed.

## Background

Thailand, like many other countries globally has been experiencing shifting patterns of drug supply and use [1-7]. Studies undertaken during the past decade suggest a number of Thai people who inject drugs (IDU) are now injecting midazolam (Thai trade name: Dormicum^®^), a legal, rapid onset, short duration benzodiazepine with potent sedative, amnestic and ventilatory depressant effects [8-10]. Midazolam is prescribed in tablet form, although it is often administered intravenously for sedation in hospital settings [9]. However, it has been reported anecdotally that some Thai physicians also prescribe midazolam for the treatment of withdrawal from opiate use [11]. A study indicated rising midazolam injection among Thai IDU, with 30% of an IDU sample reporting midazolam injection during 1999-2000 [9]. This trend was believed to coincide with the Thai government's increasing focus on drug enforcement and the declining availability and rising price of heroin in Thailand. Midazolam is much less expensive (approximately $3 USD per tablet) to acquire than heroin [8].

It has been suggested that midazolam injection, partly because of the associated amnestic effects, can result in elevated rates of risk behaviour, including syringe sharing [9]. The injection of midazolam filtrate is believed to increase risk for soft-tissue infections, gangrene, and thromboembolic events [8]. Withdrawal effects are typical of benzodiazepines and include headaches, insomnia, agitation and seizures and can be fatal [8]. Further, concerns have been expressed regarding high rates of polysubstance use among IDU who inject midazolam, prompting calls for more research on midazolam injection [9]. Given these concerns, the ongoing "drug war" in Thailand, and the paucity of research on midazolam use, we sought to examine the prevalence and correlates of midazolam injection, as well as patterns of midazolam-related polysubstance use, among a community-recruited sample of IDU in Bangkok, Thailand.

## Methods

The Mitsampan Community Research Project is a collaborative research project involving the British Columbia Centre for Excellence in HIV/AIDS (Vancouver, Canada), the Mitsampan Harm Reduction Center (Bangkok, Thailand), the Thai AIDS Treatment Action Group (Bangkok, Thailand), and Chulalongkorn University (Bangkok, Thailand). During July-August 2008, the partners undertook a cross-sectional study involving 252 community-recruited IDU. The primary aims of the study were to assess drug use and HIV risk behaviors and to assess barriers to access to healthcare among local IDU. Potential participants were recruited through peer-based outreach efforts and word of mouth. Study participants were invited to attend the Mitsampan Harm Reduction Center (MSHRC) to participate in the study. The Mitsampan Center was established in the Mitsampan neighborhood, which is home to large number of illicit drug users and low-income residents. Individuals were eligible for participation in this study and defined as an "IDU" if they reported injection of illicit drugs in the past six months. All participants provided informed consent and completed an interviewer-administered questionnaire eliciting information about demographic characteristics, drug use, HIV risk behaviour, criminal justice system exposure, and experiences with health care. All participants were given 250 Baht (approximately $7 USD) upon completion of the questionnaire. The study has been approved by the Research Ethics Boards of the University of British Columbia and Chulalongkorn University.

Using univariate statistics and multivariate logistic regression, we compared IDU who did and did not report a history of midazolam injection. Variables considered included: median age (< 36.5 years or ≥ 36.5 years), gender, education level (< secondary school vs. ≥ secondary school), heroin injection (yes vs. no), yaba (i.e., methamphetamine) injection (yes vs. no), use of drugs in combination (yes vs. no), syringe borrowing (yes vs. no), syringe lending (yes vs. no), non-fatal overdose (yes vs. no), binge drug use (yes vs. no), having had drugs planted on oneself by police (yes vs. no), incarceration (yes vs. no), compulsory treatment experience (yes vs. no), and methadone treatment (yes vs. no). Use of drugs in combination refers to use of more than one drug at the same time (i.e., not the simple use of two drugs in the same day or week). We considered experiences of drug planting by police given that this type of contact with police could potentially prompt some IDU to obtain midazolam, given that the drug can be obtained "over-the-counter" in selected pharmacies and acquiring it may involve little or no contact with the illicit drug market. This variable was ascertained by asking participants "Have police ever planted drugs on you?" Binge drug use refers to periods when drugs are used more often than usual. All behavioural variables refer to lifetime history (e.g., ever injected yaba). To examine the bivariate associations, we used the Pearson χ^2 ^test. We then examined factors independently associated with a history of midazolam injection use by fitting a multivariate logistic regression model that included all variables that were associated with midazolam injection at the *p *≤ 0.05 level in univariate analyses. All *p*-values were two-sided. We also asked participants who reported midazolam injection about the frequency of their midazolam injecting in the previous six months, and the drugs they used (if any) in combination with midazolam.

## Results

In total, 252 IDU participated in this study, including 66 (26.2%) females. The median age of participants was 36.5 years. Two hundred and thirty-eight (94.4%) participants were born in the Bangkok Metropolitan Area. In total, 170 (67.5%) participants reported that they had injected midazolam previously and, of these, 144 (81.4%, 57% of the total sample) reported daily midazolam injection in the past six months. As indicated in Table 1, in univariate analyses, factors positively associated with MSHRC use included use of drugs in combination (odds ratio [OR] = 7.53, 95% confidence interval [CI]: 4.14-13.71), syringe borrowing (OR = 1.94, 95%CI: 1.08-3.47), having drugs planted on oneself by police (OR = 3.03, 95%CI: 1.73-5.30), incarceration (OR = 2.05, 95%CI: 1.11-3.78), methadone treatment (OR = 4.29, 95%CI: 2.35-7.86), and binge drug use (OR = 2.90, 95%CI: 1.60-5.26). Younger age (OR = 0.52, 95%CI: 0.30-0.89) and female gender (OR = 0.43, 95%CI: 0.24-0.76) were negatively associated with midazolam injection. As indicated in Table 2, in multivariate analyses, midazolam injection was positively associated with use of drugs in combination (adjusted odds ratio [AOR] = 5.86; 95%CI: 2.96-11.60), binge drug use (AOR = 2.25; 95%CI: 1.09-4.63), methadone treatment (AOR = 3.12; 95%CI: 1.55-6.90), and was negatively associated with younger age (AOR = 0.43; 95%CI: 0.22-0.83). Among midazolam injectors, 65% reported using drugs in combination with other substances, with the substances most commonly used in combination with midazolam being heroin (72.3%), yaba (30.5%), methadone (7.6%), and alcohol (4.7%).

**Table 1:** Factors associated with a history of midazolam injection among IDU in MSCRP (*n *= 252)

Characteristic	Yes 67.5 (%) n = 170	No 29.8 (%) n = 82	Odds Ratio (95% CI)	*p *value
**Median age**				
< 36.5 years	76 (45)	50 (61)	0.52 (0.30 - 0.89)	0.02
≥ 36.5 years	94 (55)	32 (39)		
**Gender**				
female	35 (21)	31 (38)	0.43 (0.24 - 0.76)	< 0.01
male	135 (79)	51 (62)		
**Education**				
≥ secondary	106 (62)	43 (52)	1.50 (0.88 - 2.56)	0.14
< secondary	64 (38)	39 (48)		
**Ever injected heroin**				
yes	161 (95)	73 (89)	2.21 (0.84 - 5.79)	0.11
no	9 (5)	9 (11)		
**Ever injected *yaba *(methamphetamine)**				
Yes	109 (64)	52 (63)	1.03 (0.60 - 1.78)	0.91
no	61 (36)	30 (37)		
**Ever used drugs in combination**				
yes	142 (84)	33 (40)	7.53 (4.14 - 13.71)	**<**0.01
no	28 (16)	49 (60)		
**Binge drug use**				
yes	80 (47)	19 (24)	2.90 (1.60 - 5.26)	< 0.01
no	90 (53)	62 (76)		
**Ever borrowed used syringes**				
yes	68 (40)	21 (26)	1.94 (1.08 - 3.47)	0.03
no	102 (60)	61 (74)		
**Ever lent used syringes**				
yes	62 (36)	30 (37)	1.00 (0.58 - 1.72)	0.99
no	108 (64)	52 (63)		
**Ever overdosed**				
yes	59 (35)	16 (20)	2.19 (1.17 - 4.12)	0.02
no	111 (65)	66 (80)		
**Ever had drugs planted on you by police**				
yes	97 (57)	25 (30)	3.03 (1.73 - 5.30)	**<**0.01
no	73 (43)	57 (70)		
**Ever been in prison**				
yes	140 (82)	57 (70)	2.05 (1.11 - 3.78)	0.02
no	30 (18)	25 (30)		
**Ever been in forced drug treatment**				
yes	56 (33)	24 (29)	1.19 (0.67 - 2.11)	0.56
no	114 (67)	58 (71)		
**Ever on methadone treatment**				
yes	93 (55)	18 (22)	4.29 (2.35 - 7.86)	**<**0.01
no	77 (45)	64 (78)		

**Table 2:** Multivariate logistic regression analysis of factors associated with a history of midazolam injection in MSCRP cohort (*n *= 252)

Variable	Adjusted Odds Ratio (AOR)	95% Confidence Interval (CI)	*p *- value
**Median age**			
(< 36.5 years vs. ≥ 36.5 years)	0.43	(0.22 - 0.83)	0.01
**Gender**			
(female vs. male)	0.61	(0.29 - 1.3)	0.18
**Binge drug use**			
(yes vs. no)	2.25	(1.09 - 4.63)	0.03
**Ever used drugs in combination**			
(yes vs. no)	5.86	(2.96 - 11.60)	< 0.01
**Ever borrowed used syringes**			
(yes vs. no)	1.30	(0.64 - 2.65)	0.48
**Ever overdosed**			
(yes vs. no)	1.23	(0.55 - 2.78)	0.62
**Ever had drugs planted on you by police**			
(yes vs. no)	1.95	(0.95 - 3.98)	0.07
**Ever been in prison**			
(yes vs. no)	1.40	(0.59 - 6.27)	0.48
**Ever on methadone treatment**			
(yes vs. no)	3.12	(1.55 - 6.90)	< 0.01

## Discussion

In the present study, we found that approximately 68% of a community-recruited sample of IDU in Bangkok had injected midazolam previously. Fifty-seven percent of the sample had injected midazolam at least once a day in the past six months. Midazolam injectors were more likely to report using drugs in combination, binge drug use, and a history of methadone treatment. Midazolam injectors tended to be older, and were less likely to be female. Sixty-five percent of midazolam injectors reported use of drugs in combination, with heroin and yaba being the drugs most commonly used with midazolam.

The prevalence of midazalom injection found in the present study is much higher than most previously reported rates [9,12]. Van Griensven et al. reported a rise in self-reported midazolam injection in the previous six months, from approximately 10% in 1999 to 30% in 2000 [9], and a report from 2005 found that 73% of IDU in Bangkok had a history of midazolam injection [13]. However, while approximately 16% of the total sample in the latter study reported injecting midazolam on a daily basis in the previous month, 57% of IDU participating in our study said they injected the drug on a daily basis in the previous six months. Collectively these findings suggest that the prevalence, and more notably the intensity of midazolam injection have continued to increase steadily since 1999. It is believed that midazolam is often used as a cheaper and more accessible alternative to heroin, particularly when heroin availability declines and heroin price increases [8,9]. Previous studies have indicated that the prevalence of midazolam injecting increased following the initiation of Thailand's "War on Drugs" in February 2003 [7,14]. This initiative involved scaling up efforts to seize drugs, arrest drug dealers, and force drug users into military-style boot camps [7]. It has been reported that over 2,200 suspected drug dealers were killed via extrajudicial execution during its implementation [15]. The precise role of Thailand's drug war on the drug patterns observed herein is difficult to determine. However, previous studies have found that transitions in injection drug use as well as an initiation of, or increase in, misuse of more licit drugs may occur among drug using populations exposed to an increase in drug enforcement [16,17].

Midazolam injection was strongly associated with the use of drugs in combination and was reportedly most commonly used in combination with both heroin and methamphetamine. This raises concern regarding the potential elevated risk for overdose as a result of polysubstance use [18]; however, it is notable that while midazolam was associated with non-fatal overdose, this association did not persist in a multivariate analysis. Midazolam injection was also associated with binge drug use, which is concerning given that binge drug use has been associated with HIV infection among IDU [19]. Although concern has been expressed regarding the impact of midazolam injection on syringe sharing [9], in particular as a result of the amnestic effects of the drug, the association between syringe borrowing and midazolam injection also did not persist in our multivariate analysis.

The findings of this study have implications for harm reduction practice. First, because midazolam filtrate is highly acidic and damaging to veins, midazolam injectors are known to resort to groin injection when peripheral veins are no longer usable. Groin injection carries significant risk, including risk for deep vein thrombosis, pulmonary embolus, abscesses, and puncture of the femoral artery, vein, or nerve [20]. Therefore established harm reduction approaches specific to groin injecting should be applied in work with midazolam injectors [20], including encouraging midazolam injectors to avoid initiating groin injecting by exercising proper vein care to maintain peripheral veins, or by switching to an alternate route of drug consumption when peripheral veins are no longer accessible (i.e., non-intravenous use). Second, given that midazolam injectors frequently experience abscesses and other soft-tissue infections, efforts should be made to ensure early and appropriate care for such infections. This may require providing access to low-threshold care for soft-tissue infections. Third, given the lack of access to sterile injecting supplies in Thailand [21], efforts should be made to provide midazolam injectors with appropriate injecting supplies, including syringes and alcohol swabs. Lastly, because of the amnesic effects of midazolam and the risks associated with binge and combination drug use among midazolam injectors, educational efforts should include encouraging midazolam injectors to avoid injecting midazolam when alone.

This study has limitations. Previous studies have indicated that the majority of midazolam is distributed in Bangkok [11], and the rates of midazolam injection reported here may not generalize to other settings in Thailand. As well, the data pertaining to midazolam injection in Thailand is limited, and therefore conclusions concerning changes in the prevalence of midazolam injection should be interpreted with caution. This points further to the outstanding need for more systematic surveillance of drug use trends in Thailand, as well as data on the harms of illicit drug use, including midazolam injection. Further, the study sample was not randomly selected and therefore may not be representative of local IDU. We should also note that we relied on self-report, and therefore our data may have been affected by socially desirable responding or recall bias. Finally, we identified a number of associations with midazolam injecting, such as syringe sharing, which did not persist in multivariate analyses. Because of the limited sample size in our study, future research will be required before we can conclude that midazolam injection is not associated with elevated risk behaviour.

In summary, we found extremely high rates of midazolam injection among a cohort of Thai IDU in Bangkok. Midazolam injection was strongly associated with the use of various drugs in combination and binge drug use. Given the many adverse effects of midazolam injection, evidence-based pubic health interventions are urgently needed to reduce the harms associated with this form of drug use.

## Competing interests

The authors declare that they have no competing interests.

## Authors' contributions

TK, NKA, NF, KH, PS, KK and EW designed the study. CL conducted the statistical analyses. TK drafted the manuscript and incorporated all suggestions from co-authors. All authors made significant contributions to the conception of the analyses, interpretation of the data, and drafting of the manuscript. All authors have read and approved the final manuscript.
